# Absence of Lacteal Secretion after Parturition

**Published:** 1841-10

**Authors:** 


### ABSENCE OF LACTEAL SECRETION AFTER PARTURITION.

Dr. W. S. Sutton of Georgetown, Ky., has communicated to us the following fact: There is now in that town a lady 60 years of age, who bore and reared eight children, without her breasts discharging a drop of milk after the birth of any one of them. The glands, when she was confined with a part of them, became somewhat swollen, but no milk flowed out. She never, at any period of her life, had a nipple on either breast. D.

